# Supplementary material for: Nanostructure of nickel-promoted indium oxide catalysts drives selectivity in CO2 hydrogenation
Source: Nat Commun. 2021 Mar 30;12:1960. doi: 10.1038/s41467-021-22224-x (PMC8010022; doi:10.1038/s41467-021-22224-x)
Supplement: Supplementary file 1 — Supplementary Information [file 41467_2021_22224_MOESM1_ESM.pdf]

Supplementary Information

Nanostructure of nickel-promoted indium oxide catalysts drives selectivity in CO_2 hydrogenation

Frei *et al.*

Table of Contents

Supplementary Methods 2
Catalyst preparation 2
Catalyst characterization 3
Product analysis by gas chromatography 6
Modelling of Ni atoms on In_2O_3(111) 7
Assessment of metal-support interactions 8
Supplementary Figures 10
Supplementary Tables 23
Supplementary References 33

Supplementary Methods

Catalyst preparation. To obtain pure In_2O_3, $In(NO_3)_3{\cdot}xH_2O$ (15.5 g, Sigma-Aldrich, 99.99%) was dissolved in deionized water (235 cm^3). A solution of aqueous ammonia (120 cm^3, 25 wt.%, Sigma-Aldrich) and ethanol (350 cm^3, Sigma-Aldrich, >98%) was prepared and added dropwise to the former solution under magnetic stirring at ambient temperature to reach a pH value of 9.2. The resulting slurry was aged for 1 h. Then, the solid was separated by high-pressure filtration, washed with deionized water (3 times, *ca.* 500 cm^3 each time), and dried in a vacuum oven (2 kPa, 323 K, 12 h). Thereafter, the powder was calcined for 3 h at 773 K (heating rate = 2 K min^{-1}) in static air. Ni-In_2O_3 catalysts were prepared by coprecipitation (CP) adding $In(NO_3)_3{\cdot}6.9H_2O$ (3.5 g, Sigma-Aldrich, 99.99%) and $Ni(NO_3)_2{\cdot}6H_2O$ (24.6, 37.0, 49.3, or 123 mg, Sigma-Aldrich, ≥97.0%) to deionized water (50 cm^3) in a round-bottomed flask (250 cm^3). Na_2CO_3 (10.0 g) was dissolved in deionized water (100 cm^3) and added dropwise (*ca.* 25 cm^3, 3 cm^3 min^{-1}) to the metals' solution under stirring at room temperature to reach a pH of 9.2. After aging the resulting slurry for 1 h, deionized water was added (50 cm^3). Then, the precipitate was recovered by high-pressure filtration, washed with deionized water (3 times, 500 cm^3 each time), and dried in a vacuum oven (1.5 kPa, 323 K, 1.5 h), followed by calcination for 3 h at 573 K (2 K min^{-1}) in static air. To produce Ni-In_2O_3 catalysts by dry impregnation (DI), a round-bottomed flask (25 cm^3) was loaded with $Ni(NO_3)_2{\cdot}6H_2O$ (13.8, 27.6, 68.6, 137, 274, 410, or 548 mg) and deionized water (0.60 g). After adding the support (1.00 g) and 5 stainless-steel spheres (radius = 1.5 mm), the flask was rotated (*ca.* 45 rpm) using a Büchi R-114 rotary evaporator at room temperature and pressure. After 12 h, the pressure was lowered to 2 kPa and the temperature raised to 333 K for 1 h to allow for the evaporation of the solvent. The resulting solid was calcined for 3 h at 623 K (2 K min^{-1}) in static air. Mixed aluminum-indium oxides used as supports (denoted as *n*InAl, where *n* is the molar content of indium) were prepared following a literature protocol.[1]

$In(NO_3)_3·6.9H_2O$ (5.56, 3.72, 1.86, 1.86, or 0.93 g) and $Al(NO_3)_3·9H_2O$ (1.88, 3.75, 5.63, 16.9, or 17.8 g, Merck, 99% metals basis) were dissolved in deionized water (200 cm^3). Then a solution of aqueous ammonia (100 cm^3) and ethanol (100 cm^3) was prepared and added dropwise (*ca.* 2 cm^3 min^{-1}) to the former solution under vigorous magnetic stirring (*ca.* 750 rpm) until reaching a pH value of 8.5. The obtained precipitate was separated by high-pressure filtration, dried in a vacuum oven (1.5 kPa, 323 K, 1.5 h), and calcined for 6 h at 773 K (5 K min^{-1}) in static air. The attained solids were used to prepare catalysts with 5wt.% nickel content by DI as detailed above.

Catalyst characterization. Nitrogen sorption at 77 K was conducted using a Micromeritics TriStar II analyzer. Prior to the measurements, samples were degassed at 573 K under vacuum for 3 h. The total surface area (S_{BET}) was determined using the BET model. X-ray fluorescence spectroscopy (XRF) was performed using an Orbis Micro-EDXRF spectrometer equipped with a Rh source operated at 35 kV and 500 μA and a silicon drift detector. High-pressure temperature-programmed reduction with hydrogen (H_2-TPR) was carried out in a Micromeritics Autochem HP II analyzer. Prior to the analysis, samples (100 mg) were dried *in situ* in flowing Ar (40 cm^3_{STP} min^{-1}) at 393 K (5 K min^{-1}) for 1 h, followed by cooling to 273 K (5 K min^{-1}). Then the pressure was raised to 5 MPa, the inlet flow changed to 5 mol% H_2 in He (20 cm^3_{STP} min^{-1}), and the temperature was increased to 1073 K (5 K min^{-1}), while analyzing the effluent stream by mass spectrometry using a Pfeiffer Omnistar GSD 320 instrument monitoring the mass-to-charge ratios (*m*/*z*) 2 and 18. Temperature-programmed desorption of carbon monoxide (CO-TPD) was performed in the same unit as H_2-TPR, using an equivalent amount of sample (100 mg). Following a pretreatment at 553 K (5 K min^{-1}) in flowing H_2 (5 mol% H_2/He) at 5 MPa, the sample was cooled to 273 K (10 K min^{-1}) and the probe molecule was adsorbed at 0.1 MPa over 30 min in a flowing CO (10 cm^3 min^{-1}, 5 mol%

CO in Ar). Then, the gas flow was changed to Ar (50 cm^3_{STP} min^{-1}) and the temperature was increased to 1073 K (5 K min^{-1}), while analyzing the effluent stream by mass spectrometry recording *m*/*z* 28 and 44. Near ambient-pressure Diffuse reflectance Fourier transform infrared spectroscopy of adsorbed CO (CO-DRIFTS) was measured in a Bruker Vertex 70 spectrometer, equipped with a Harrick Praying Mantis mirror assembly and a reaction chamber, in the range of 4000-650 cm^{-1}, with 2 cm^{-1} optical resolution and accumulation of 300 scans. The sample was diluted 5 times by weight with silicon powder (ABCR-Chemicals, 99.995%) and placed in the cell, followed by heating to 553 K for 30 min (10 K min^{-1}) in flowing H_2 (10 cm^3_{STP} min^{-1}, 5 mol% H_2/He) at 5 MPa of pressure. Thereafter, the pressure was reduced to 0.1 MPa, the inlet flow changed to Ar (10 cm^3_{STP} min^{-1}) and the sample cooled to room temperature. Adsorption of carbon monoxide was conducted in flowing CO (10 cm^3_{STP} min^{-1}, 5 mol% CO/Ar) for 30 min with subsequent Ar purging (10 cm^3_{STP} min^{-1}) for 30 min. Spectra were recorded before and after the chemisorption step, and the former was used as a background for the latter. XRD was measured at the Materials Science X04SA beam line at the Swiss Light Source (SLS, Villigen, Switzerland) in an experimental setup described in detail elsewhere.[2] Samples were sealed in quartz capillaries with a 0.3-mm inner diameter. To prepare used catalysts, capillaries containing the catalyst were placed in the reactor used for CO_2 hydrogenation, exposed to the typical reaction conditions, and welded shut after the test. Measurements were carried out in transmission mode with a 20-keV beam and a 70 × 70 μm^2 spot size probing the center of the capillary. Diffraction data was recorded using the Mythen II microstrip detector in the range of 0-30° 2θ. Nickel K-edge X-ray absorption spectroscopy (XAS) was measured at the SuperXAS beamline at the SLS.[3] The incident beam was provided by the 2.9 T super-bent source and was collimated by a Si-coated mirror at 2.5 mrad and focused by a toroidal Rh-coated mirror to 200 × 500 μm. The energy was selected by a Si(111) channel-cut monochromator,[4] and calibrated using a nickel foil (8.3328 keV), which was

measured simultaneously with the specimen of interest. Samples were transferred from the reactor to quartz capillaries under inert atmosphere. The 15Ni-In_2O_3 catalyst was diluted with boron nitride to avoid self-absorption. The NiO reference was measured in transmission mode as a pellet diluted with cellulose. Three 15-cm long ionization chambers filled with nitrogen at 1.5 bar were used to monitor the incident beam intensity,[4] transmission through the sample, and the reference metallic Ni foil. A Pips diode was used to measure the fluorescence signal from the catalysts. Nickel K-edge XAS data were calibrated and averaged with in-house developed ProXAS software and further analyzed using the Demeter software.[5] k^3-weighted extended X-ray absorption fine structure (EXAFS) spectra were fitted in R-space in the k-range of 3-9 Å^{-1} (for 1Ni-In_2O_3) or 3-11 Å^{-1} (for 15Ni-In_2O_3) and R-range of 1-3 Å. An amplitude reduction factor of 0.82 was determined by fitting of the EXAFS spectrum of the metallic Ni foil. The scattering paths for fitting of all data were produced using known crystallographic structures of metallic Ni, NiO, and Ni_3In. Scanning transmission electron microscopy coupled to energy-dispersive X-ray spectroscopy (STEM-EDX) as well as high resolution transmission electron microscopy (HRTEM) were performed using a Talos F200X instrument operated at 200 kV and equipped with a FEI SuperX detector and an aberration-corrected JEM-ARM300F microscope (GrandARM, JEOL), operated at 300 kV with a cold-field electron gun emitter ($\Delta E \approx 0.35$ eV). Volumetric chemisorption of CO_2 was carried out in a Micromeritics 3Flex Chemi analyzer. Prior to the analysis, the sample (200 mg) was reduced *in situ* at 573 K in pure H_2 for 1 h. Thereafter, the reactor was cooled down to 318 K and CO_2 (PanGas, 99.9%) was dosed in increments of 13.3 kPa until a pressure of 93.3 kPa was reached. To distinguish between weak and strong adsorption, the dual-isotherm method was applied, where the first isotherm includes strong and weak adsorption and the second isotherm, recorded after evacuation of the catalyst at analysis temperature, only relates to weakly adsorbed molecules.[6]

Product analysis by gas chromatography. The gas composition of the reactor outlet was analyzed by gas chromatography, making use of an internal standard and response factors. The response factors F_i for each compound i, respective to the internal standard (C_2H_6), in the gas chromatography analysis were determined using the equation:

$$F_i = \frac{A_i / \dot{n}_i^{\text{in}}}{F_{\text{C}_2\text{H}_6} / \dot{n}_{\text{C}_2\text{H}_6}^{\text{in}}} \tag{1}$$

where A_i is the integrated area determined for the peak of compound i in the gas chromatogram and $\dot{n}_i^{\text{in}}$ corresponds to its known molar flow rate at the reactor inlet. The response factors of the analytes i were calculated as the average of 5 points around the expected concentrations of the respective analyte. Under reaction, the unknown outlet molar flow rate $\dot{n}_i^{\text{out}}$ was determined using the equation:

$$\dot{n}_i^{\text{out}} = \frac{A_i \times F_i}{A_{\text{C}_2\text{H}_6}} \times \dot{n}_{\text{C}_2\text{H}_6}^{\text{in}}, \ \text{mol}_i \ \text{h}^{-1} \tag{2}$$

Conversion (X_i), selectivity (S_i), and production rate (r_i) were calculated applying the equations:

$$X_i = \frac{\dot{n}_i^{\text{in}} - \dot{n}_i^{\text{out}}}{\dot{n}_i^{\text{in}}} \times 100, \ \% \tag{3}$$

$$S_i = \frac{\dot{n}_i^{\text{in}} - \dot{n}_i^{\text{out}}}{\dot{n}_{\text{CO}_2}^{\text{in}} - \dot{n}_{\text{CO}_2}^{\text{out}}} \times 100, \ \% \tag{4}$$

$$r_i = \frac{\dot{n}_i^{\text{in}} - \dot{n}_i^{\text{out}}}{w_{\text{cat}}}, \ \text{mol}_i \ \text{h}^{-1} \ \text{g}_{\text{cat}}^{-1} \tag{5}$$

where w_{cat} is the amount of catalyst. The methanol space-time yield (*STY*) is the product of r_{MeOH} and the molar weight of methanol (32.04 g mol^{-1}). Data reported correspond to the average of the 4 measurements preceding a specific time-on-stream, or to the average of 7

measurements collected during each individual condition when temperature or gas flows were altered. The carbon balance was determined for each experiment according to equation:

$$\varepsilon_C = \left(1 - \frac{\dot{n}^{out}_{CO_2} + \dot{n}^{out}_{MeOH} + \dot{n}^{out}_{CO}}{\dot{n}^{in}_{CO_2} + \dot{n}^{in}_{MeOH}}\right) \times 100,\ \% \tag{6}$$

and was found to be always within 3%.

Modelling of nickel atoms on $In_2O_3(111)$. The behavior of nickel atoms on In_2O_3 (Ni_x-In_2O_3, $x = 1, 2, 3, 4, 25, 27, 36$) was modeled starting by two *ansatz*: nickel layers covering the In_2O_3 surface, detailed in the main manuscript, and nickel single-site entities, such as those identified earlier for Pd-doped In_2O_3.[7] For the second model, 1-4 nickel atoms were placed on pristine $In_2O_3(111)$ adsorbed between three oxygen atoms on all symmetrically inequivalent positions throughout the surface, as well as in three random positions around the protrusion (**Supplementary Figure 9**).[7] The stability of these structures per nickel atom was evaluated taking NiO(s), H_2O(g), H_2(g), and the pristine In_2O_3 surface as references, based on the equations:

$$\text{NiO} + \frac{x+v}{x}\text{H}_2 - \frac{x+v}{x}\text{H}_2\text{O} + \frac{1}{x}\text{In}_2\text{O}_3 \rightarrow \frac{1}{x}\text{Ni}_x\text{-In}_2\text{O}_{3-\frac{v}{120}} \tag{7}$$

$$E = \frac{1}{x}E_{\text{Ni}_x\text{-In}_2\text{O}_{3-\frac{v}{120}}} - E_{\text{NiO}} - \frac{x+v}{x}E_{\text{H}_2} + \frac{x+v}{x}E_{\text{H}_2\text{O}} - \frac{1}{x}E_{\text{In}_2\text{O}_3} \tag{8}$$

where v is the number of oxygen vacancies created on the most external layer of $In_2O_3(111)$, which originally contained 120 oxygen atoms, and x the number of nickel atoms placed on top of it. Single-atom entities (Ni_1-In_2O_3) were largely unstable, with the exception of a nickel atom adsorbed on bridge sites around the protrusion, either between two (−0.56 eV) or three (−0.26 eV) oxygen atoms. The most stable of the other nuclearities contained two nickel atoms on bridge sites around the protrusion (Ni_2-In_2O_3). Surfaces with 3 and 4 Ni atoms were less stable. The subsequent formation of oxygen vacancies around the protrusion was thermoneutral

for one oxygen vacancy with one nickel atom (+0.06 eV, Ni_1-In_2O_3) and endothermic for two nickel atoms (+0.53 eV, Ni_2-In_2O_3).

Assessment of metal-support interactions. Bulk metals, intermetallics, and oxides were modeled from their stable structures at room conditions, taking the references outlined in **Supplementary Table 8** as initial guesses. To obtain the most accurate geometries, all bulk structures were fully relaxed with a higher kinetic energy cutoff (800 eV) and a denser *k*-mesh than for pure In_2O_3(111). Final energies were attained through single-point calculations with a kinetic energy cutoff of 500 eV over optimized structures. Formation energies were then calculated using isolated gas-phase metal atoms, bulk oxide support, and gas-phase H_2, O_2, and H_2O molecules as references, and reported respective to the total number of metal atoms. For bulk metals, the following equations were used:

$$M_{(g)} \rightarrow M_{(s)} \tag{9}$$

$$E_{f(M)} = E_{M(s)} - E_{M(g)} \tag{10}$$

where $E_{M(s)}$ is the bulk energy by metal atom and $E_{M(g)}$ is the energy of an isolated gas-phase metal atom. Therefore, $E_{f(M)}$ corresponds to the opposite of the cohesive energy. The formation of metal oxides from gas-phase metals and O_2 is reported by the number of metal atoms:

$$aM_{(g)} + {}^{b}/_{2}O_{2(g)} \rightarrow M_aO_{b(bulk)} \tag{11}$$

$$E_{f(M_aO_b)} = [E_{M_aO_b(bulk)} - aE_{M(g)} - {}^{b}/_{2}E_{O_2(g)}]/a \tag{12}$$

where $E_{M_aO_b(bulk)}$ is the energy of a formula unit of bulk metal oxide and $E_{O_2(g)}$ is the energy of the O_2 molecule. The formation of intermetallics and H_2O from the metal oxide, the deposited gas-phase metal and H_2 is analyzed with the following equations:

$$xbH_{2(g)} + xM_aO_{b(bulk)} + ayM'_{(g)} \rightarrow aM_xM'_{y(bulk)} + xbH_2O_{(g)} \tag{13}$$

$$E_{f(M_xM'_y)} = [E_{M_xM'_y(bulk)} + {}^{xb}/_{a}\,E_{H_2O(g)} - {}^{x}/_{a}E_{M_aO_b(bulk)} - yE_{M'(g)} - {}^{xb}/_{a}\,E_{H_2(g)}]/y \tag{14}$$

where M and M' represent the metal of the oxide and the deposited metal, $E_{M_xM'_y(bulk)}$ is the energy of the alloy, and $E_{H_2O(g)}$ and $E_{H_2(g)}$ are the energies of a water and a H_2 molecule, respectively. The final $E_{f(M_xM'_y)}$ energy is reported by the number of deposited metal atoms.

Supplementary Figures

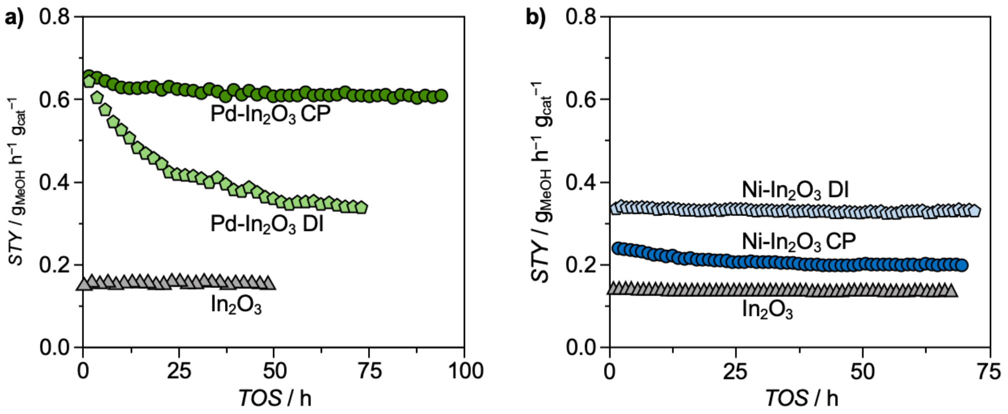

Supplementary Figure 1. Evolution of methanol space-time yield (*STY*) with time-on-stream (*TOS*) for **a)** palladium- (0.75 wt.%) and **b)** nickel-promoted (1 wt.%) In_2O_3 prepared by coprecipitation (CP) and dry impregnation (DI), with pure In_2O_3 serving as a reference. Reaction conditions: $T = 553$ K, $P = 5$ MPa, molar $H_2:CO_2 = 4$, and $WHSV = 24{,}000$ $cm_{STP}^3\ h^{-1}\ g_{cat}^{-1}$.

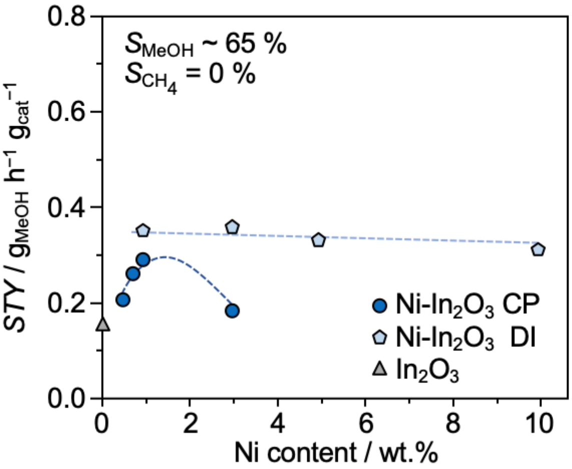

Supplementary Figure 2. Methanol space-time yield (*STY*) as a function of the nominal nickel content for nickel-promoted In_2O_3 catalysts prepared by CP and DI, with pure In_2O_3 serving as a reference. Reaction conditions: $T = 553$ K, $P = 5$ MPa, molar H_2:CO_2 = 4, and $WHSV = 24{,}000\ cm_{STP}^3\ h^{-1}\ g_{cat}^{-1}$.

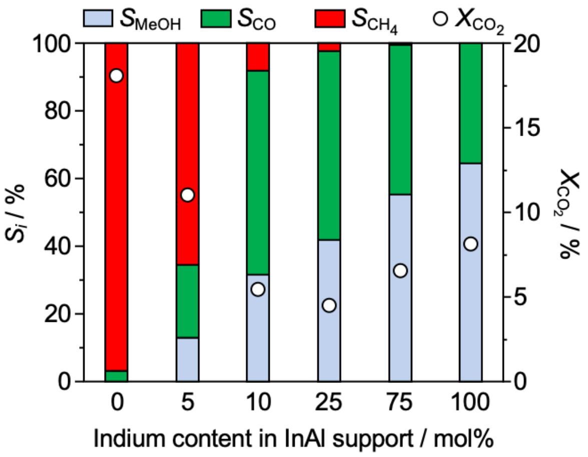

Supplementary Figure 3. Evolution of product selectivity and CO_2 conversion as a function of the indium content in InAl mixed-oxide supports containing 5 wt.% of nickel. Reaction conditions: T = 553 K, P = 5 MPa, molar H_2:CO_2 = 4, and $WHSV$ = 24,000 cm_{STP}^3 h^{-1} g_{cat}^{-1}.

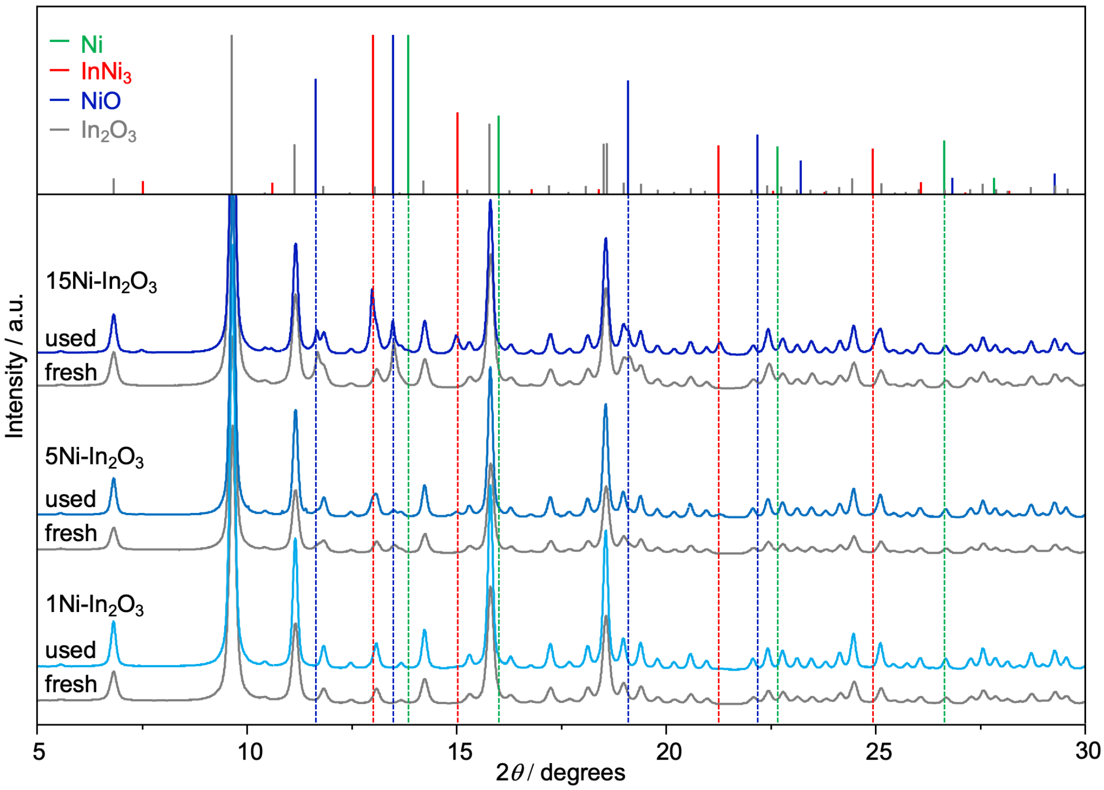

Supplementary Figure 4. XRD patterns collected in monochromatic light (λ = 0.49292 Å) of nickel-promoted In_2O_3 catalysts in fresh and used forms. The number in the sample code denotes the nickel content. Expected locations and normalized intensities of all detected reflections are shown at the top (ICSD: 169420, 59439, 92128, 646089). Reaction conditions: T = 553 K, P = 5 MPa, molar H_2:CO_2 = 4, $WHSV$ = 24,000 cm_{STP}^3 h^{-1} g_{cat}^{-1}, and time-on-stream = 16 h.

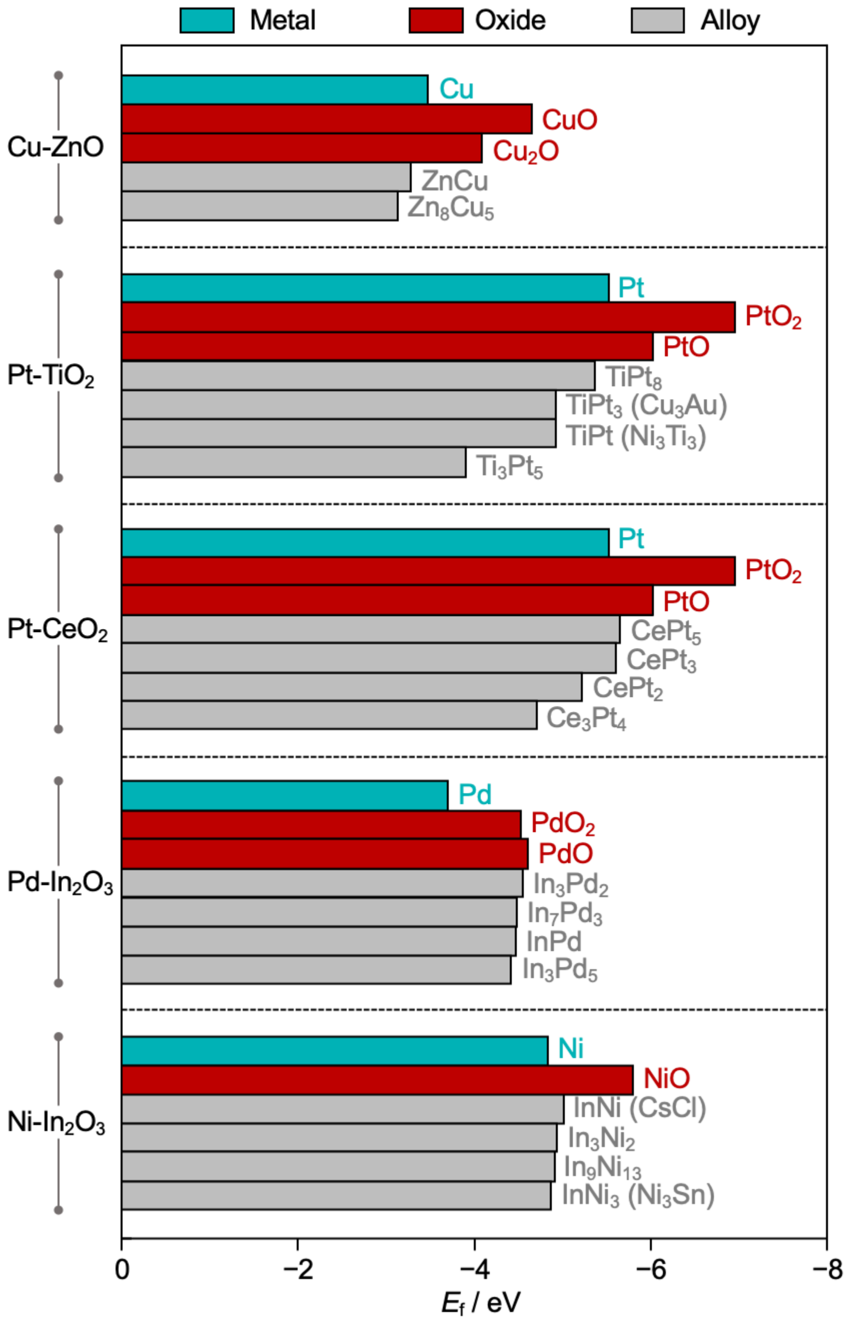

Supplementary Figure 5. Formation energy, E_f, of metal, metal oxide, and intermetallic phases with the metal of the oxide carrier for different catalysts exhibiting metal-support interactions. Deposition of metal atoms on oxide surfaces *via* impregnation and reduction of metal salts can result in the formation of either metallic or oxidic phases, depending on the reduction conditions and the relative stability of different phases. The support can also influence the nature of the metal phase deposited, including the possibility of forming intermetallic covalent bonds.[8] This effect was analyzed for Ni-In_2O_3 and other known CO_2 hydrogenation systems (Cu-ZnO, Pt-TiO_2, Pt-CeO_2, and Pd-In_2O_3) by studying the formation energy of the corresponding metals, metal oxides, and intermetallic phases between bulk and deposited metals. Results are summarized in **Supplementary Table 8**. Calculated formation energies for metals and oxides are in good agreement with experimental standard enthalpies of formation (**Supplementary Figure 6** and **Supplementary Table 9**).

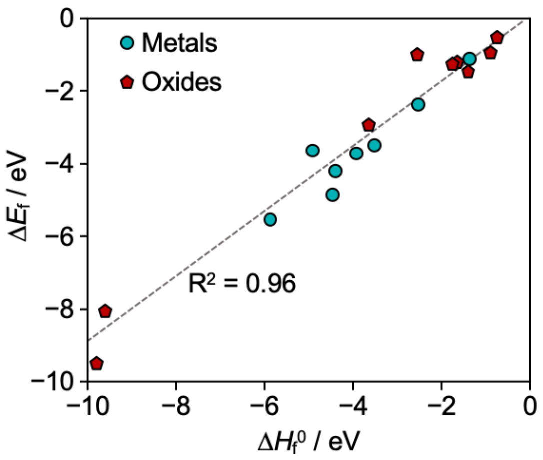

Supplementary Figure 6. Comparison between calculated formation energy, ΔE_f, and experimental standard enthalpy of formation, $\Delta H_\mathrm{f}{}^0$, for metals and metal oxides.

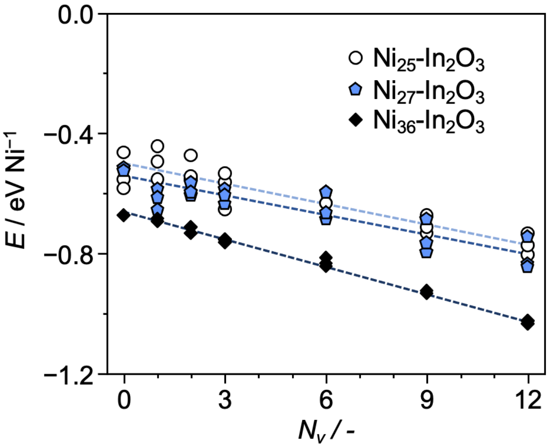

Supplementary Figure 7. Stability of selected Ni_x-In_2O_3 models as a function of the number of oxygen vacancies (N_v) created on In_2O_3.

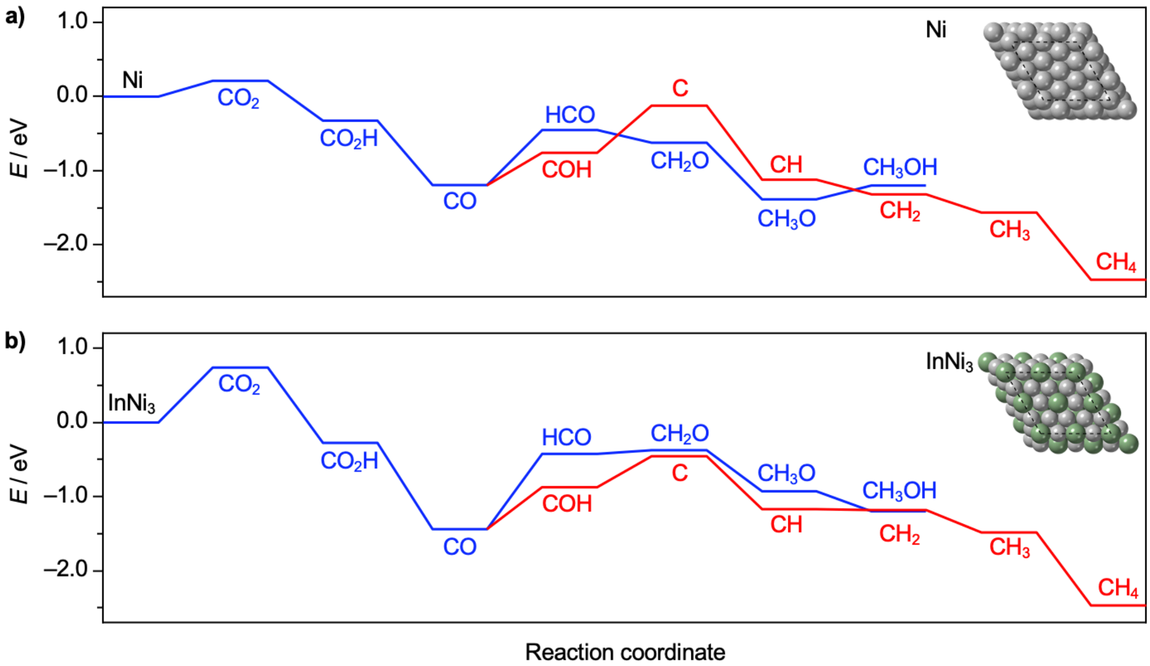

Supplementary Figure 8. Energy profile for CO_2 hydrogenation to methanol (blue) and methane (red) on **a)** Ni(111) and **b)** $InNi_3$(111).

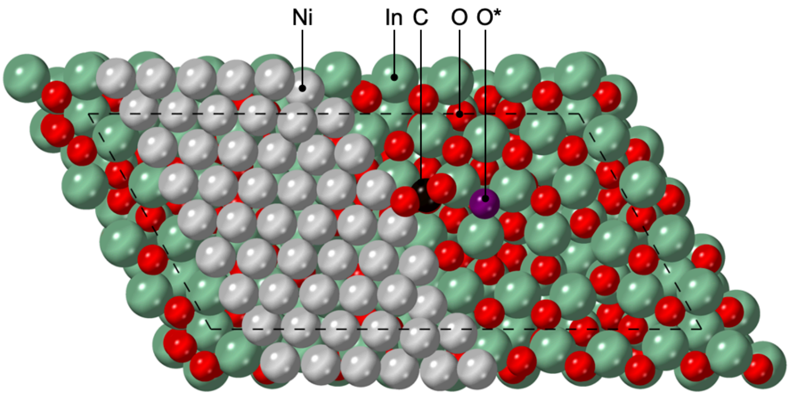

Supplementary Figure 9. CO_2 adsorption at the boundary between undoped In_2O_3(111) and a strip of Ni(111) equivalent to Ni_{36}-In_2O_{3-v} using a model was created on a 2×1 expansion of the In_2O_3 unit cell (dashed line). The CO_2 adsorption energy increases from –0.56 to –0.52 eV when an oxygen vacancy is created *via* the abstraction of the O* atom marked in purple. For nickel-free In_2O_3(111), the adsorption energy is –0.56 eV. Hence, the boundary between the active site on In_2O_3(111) and the $InNi_3$ alloy layer may also be active for CO_2 hydrogenation.

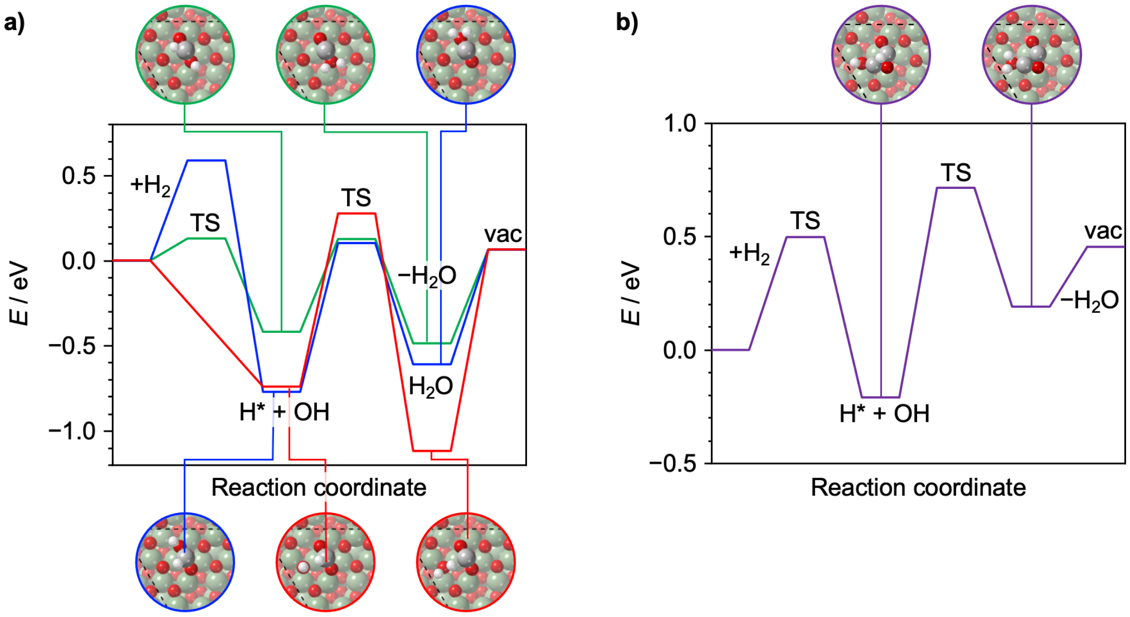

Supplementary Figure 10. Vacancy formation mechanism on (**a**) Ni_1-In_2O_3 and (**b**) Ni_2-In_2O_3. The process starts when a H_2 molecule dissociates heterolytically on a Ni-O pair, where a hydride binds to a nickel atom and a proton moves to one of the three different oxygen atoms at the protrusion. Then, water is formed and desorbs. Green and blue paths represent dissociations that are inequivalent by symmetry. H_2 dissociation may have an activation barrier of 0.13 or 0.59 eV if the proton accommodates onto an oxygen atom bound to a nickel atom. After that, both paths reach the formation of water with activations energies of 0.55 (green) and 0.87 (blue) eV. Water is finally desorbed leading to an oxygen vacancy. In a third path (red), H_2 dissociation is barrierless and occurs when a proton binds to an oxygen atom not bound to a nickel atom. However, water formation has an activation energy of 1.02 eV. Hence, oxygen vacancy generation is more unfavorable following this path. In all cases, the barrier is much lower than for clean In_2O_3, on which the first vacancy is formed by homolytic H_2 dissociation leading to two surface OH groups with a barrier of 1.30 eV. On Ni_2-In_2O_3 (purple) in **b)**, the activation energy for heterolytic H_2 dissociation and water formation are 0.50 and 0.92 eV, respectively. These values are in line with the results of Ni_1-In_2O_3. The formation of the first vacancy on Ni_2-In_2O_3 is uphill by 0.52 eV. Overall, the presence of nickel facilitates H_2 dissociation on In_2O_3 and, due to the reaction conditions applied, oxygen vacancy formation is facile on both Ni_1-In_2O_3 and Ni_2-In_2O_3.

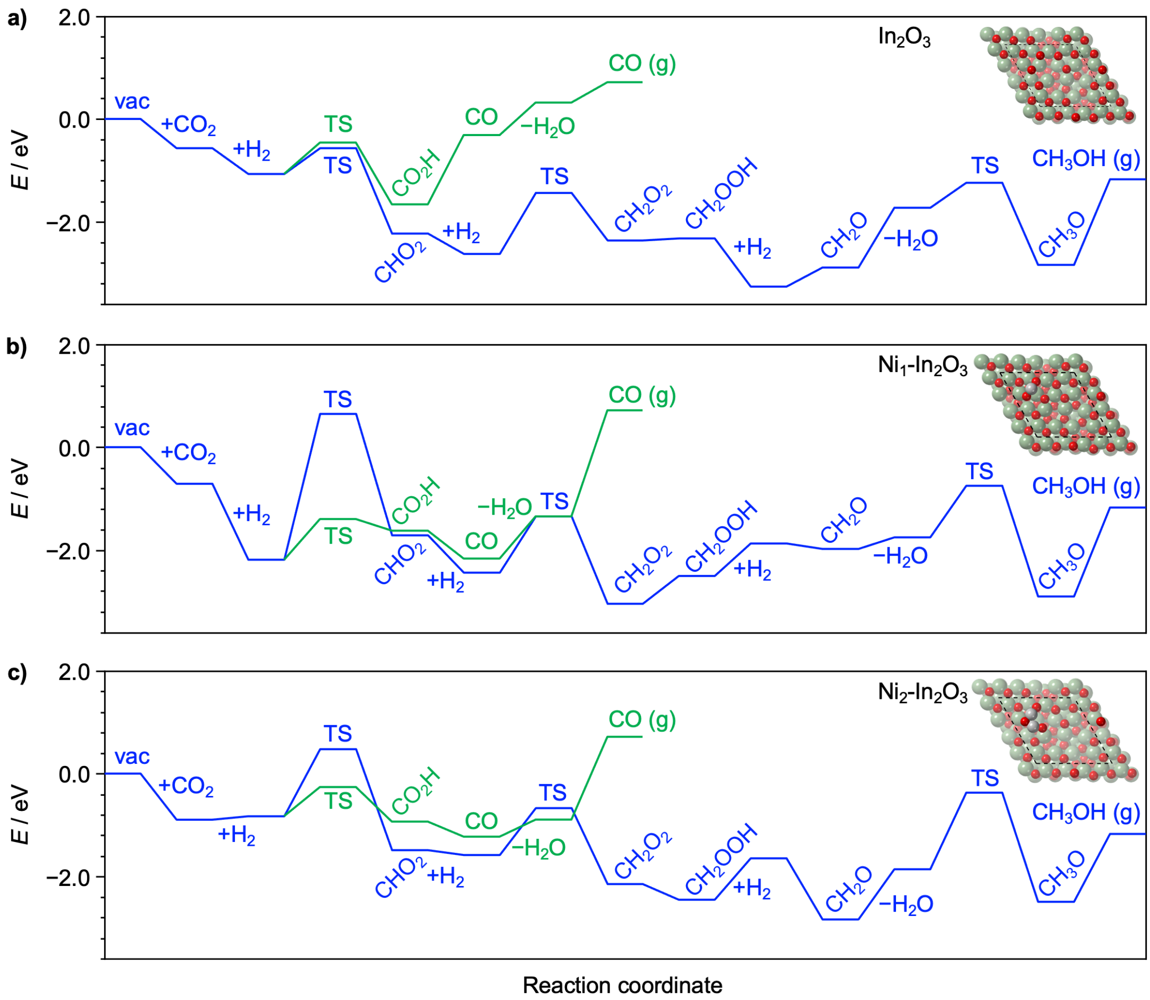

Supplementary Figure 11. CO_2 hydrogenation mechanism to methanol (blue) and CO (green) on **a**) In_2O_3, **b)** Ni$_1$-In_2O_3, and **c)** Ni$_2$-In_2O_3. On all Ni$_x$-In_2O_3 surfaces, the process starts when CO_2 adsorbs with an oxygen atom occupying the vacancy and the carbon atom binding an oxygen atom of the protrusion linked to a nickel atom. CO production encompasses the transfer of a hydrogen atom to the oxygen of CO_2 leading to CO_2H, followed by further hydrogenation and cleavage. In the case of methanol synthesis, hydrogenation takes place at the carbon atom of CO_2 attaining HCO_2. Then, a second H_2 molecule dissociates heterolytically on a Ni-O pair and proton transfer occurs forming H_2CO_2, which is further hydrogenated to CH_2OOH. A third heterolytic H_2 dissociation allows protonation of the hydroxyl group of CH_2OOH, leading to CH_2O and H_2O, the latter of which desorbs. After that, methoxide (H_3CO) is produced through transfer of the hydrogen species adsorbed on nickel to the carbon atom of H_2CO. Finally, H_3CO is hydrogenated at the oxygen atom and methanol desorbs. In contrast to unpromoted In_2O_3, hydrogenation leading to methanol has a high energy barrier on the Ni$_x$-In_2O_3 sites. As a result, Ni-doped ensembles are highly selective to CO. The RWGS is more promoted on the on Ni$_2$-In_2O_3 sites as adsorbed CO_2 and H_2 are slightly less stable than on Ni$_1$-In_2O_3, resulting in a flatter energy profile.

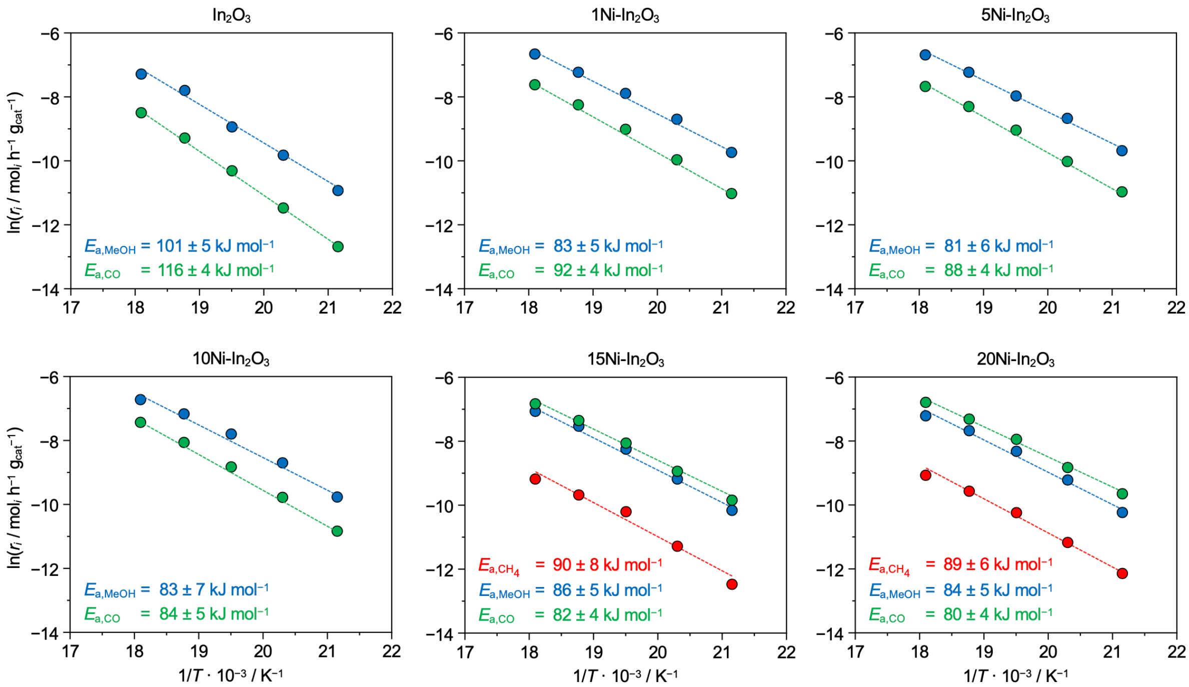

Supplementary Figure 12. Linear fittings of catalytic data collected at variable temperature to extract apparent activation energies (E_a) of MeOH, CO, and CH_4 formation over Ni-In_2O_3 catalysts with 0-20 wt.% Ni content. Reaction conditions: P = 5 MPa, molar H_2:CO_2 = 4, and $WHSV$ = 24,000 cm_{STP}^3 h^{-1} g_{cat}^{-1}.

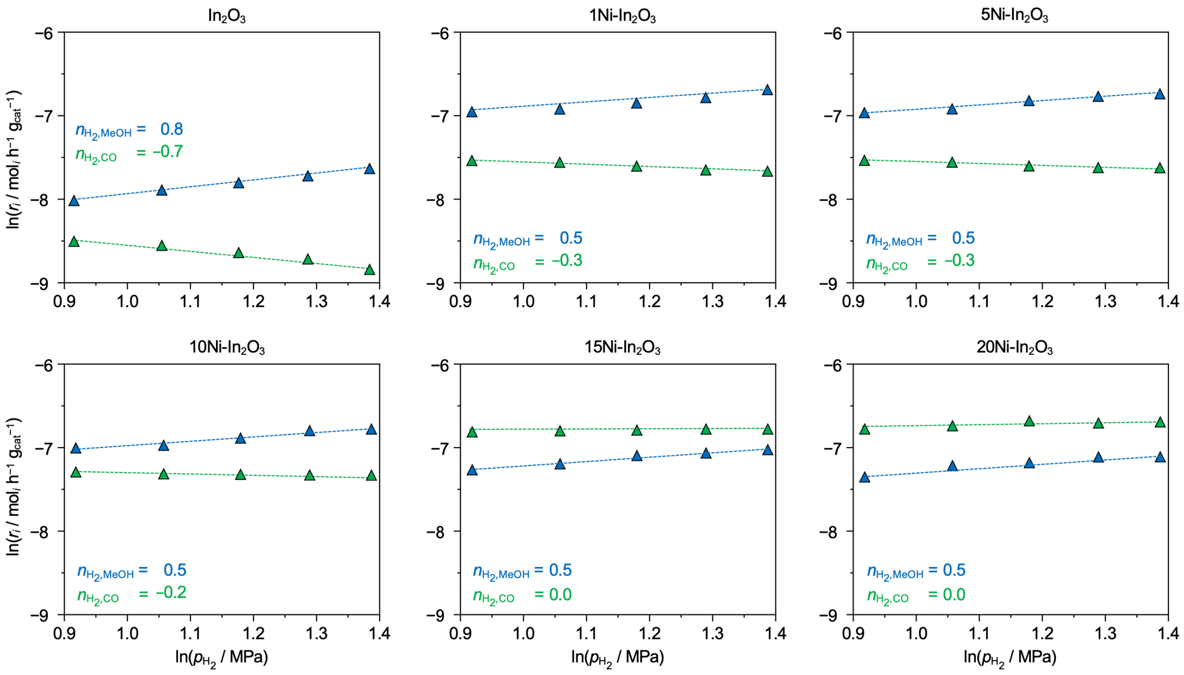

Supplementary Figure 13. Linear fittings catalytic data collected at variable partial pressures of H_2 (p_{H_2}) to determine apparent reaction orders with respect to hydrogen (n_{H_2}) for methanol synthesis and the RWGS reaction over Ni-In_2O_3 catalysts with 0-20 wt.% Ni content. Reaction conditions: T = 553 K, P = 5 MPa, and $WHSV$ = 24,000 cm_{STP}^3 h^{-1} g_{cat}^{-1}.

Supplementary Tables

Supplementary Table 1. Synthetic approach and compositional and structural characterization data for all the materials discussed in the main manuscript.

Catalyst	Synthesis method[a]	Nominal Ni content [wt.%]	Measured Ni content [wt.%]	V_{pore}[c] [cm^3 g^{-1}]	S_{BET}[c] [m^2 g^{-1}]
In_2O_3	P	-	-	0.31	75
0.5Ni-In_2O_3	CP	0.5	0.44	0.26	128
0.75Ni-In_2O_3	CP	0.75	0.72	0.38	128
1.0Ni-In_2O_3	CP	1.0	0.94	0.43	142
2.5Ni-In_2O_3	CP	2.5	2.51	0.39	112
0.5Ni-In_2O_3	DI	0.5	0.54	0.31	72
1Ni-In_2O_3	DI	1	1.03	0.29	70
2.5Ni-In_2O_3	DI	2.5	2.61	0.29	69
5Ni-In_2O_3	DI	5.0	5.21	0.30	75
10Ni-In_2O_3	DI	10.0	10.07	0.33	72
15Ni-In_2O_3	DI	15.0	16.10	0.22	69
20Ni-In_2O_3	DI	20.0	21.31	0.20	61
5Ni-Al_2O_3	DI	5	5.35	0.52	152
5Ni-5InAl	DI	5	5.20	0.67	172
5Ni-10InAl	DI	5	5.07	0.50	136
5Ni-25InAl	DI	5	5.09	0.45	97
5Ni-75InAl	DI	5	5.11	0.37	89

[a] P: precipitation, DI: dry impregnation, CP: coprecipitation. [b] XRF. [c] N_2 sorption.

Supplementary Table 2. Analysis of the H_2-TPR data presented in **Figure 2a** of the main manuscript.

Sample	NiO signal [a.u.]	In_2O_3 signal [a.u.]	NiO normalized[a] [-]	In_2O_3 normalized[b] [-]	In_2O_3/NiO ratio [-]
In_2O_3	0	0.000223	0	1	-
5Ni-Al_2O_3	0.000500	0	1	0	0
1Ni-In_2O_3	0.000078	0.000673	0.1583	3.0097	8.53
5Ni-In_2O_3	0.000333	0.000773	0.6663	3.4570	2.32
10Ni-In_2O_3	0.001104	0.000863	2.2048	3.8582	0.78
15Ni-In_2O_3	0.002248	0.001158	4.4892	5.1805	0.51

[a] Normalized to 5Ni-Al_2O_3. [b] Normalized to In_2O_3.

Supplementary Table 3. Results of nickel K-edge EXAFS analysis for the data presented in **Figure 4c** of the main manuscript. ΔE is edge energy shift, and R_{eff} and R correspond to the initial and fitted length of each scattering path used for refinement.

Sample	Scattering path	ΔE [eV]	Number of neighbors	R_{eff} [Å]	R [Å]	Debye-Waller factor [$Å^2$]
1Ni-In_2O_3 used	Ni-Ni	−5 ± 4	1.4 ± 1.0	2.65	2.59 ± 0.05	0.053 ± 0.005
	Ni-In	−5 ± 4	4.5 ± 2.0	2.65	2.62 ± 0.03	0.053 ± 0.005
5Ni-In_2O_3 used	Ni-Ni	−7 ± 2	2.4 ± 0.9	2.65	2.54 ± 0.02	0.008 ± 0.003
	Ni-In	−7 ± 2	4.1 ± 0.9	2.65	2.56 ± 0.02	0.008 ± 0.003
15Ni-In_2O_3 used	Ni-Ni	−5 ± 1	3.7 ± 0.7	2.65	2.58 ± 0.01	0.0095 ± 0.001
	Ni-In	−5 ± 1	4.9 ± 0.8	2.65	2.62 ± 0.01	0.0095 ± 0.001

Supplementary Table 4. Compositional, porosity, and oxygen vacancy characterization data of unpromoted and nickel-promoted In_2O_3 and the reference Ni-Al_2O_3.

Sample	Ni content[a]	S_{BET}[b]	CO_2 uptake[c]		Accessible In_2O_3 surface[d]
	[wt. %]	[m^2 g^{-1}]	[cm^3 g^{-1}]	[cm^3 m^{-2}]	[%]
In_2O_3	-	75	1.5	0.0200	100
1Ni-In_2O_3	1.03	70	1.3	0.0186	93
5Ni-In_2O_3	5.21	74	1.2	0.0162	81
10Ni-In_2O_3	10.07	71	0.9	0.0127	63
15Ni-In_2O_3	16.10	69	0.5	0.0072	36
5Ni-Al_2O_3	5.35	152	0.2	0.0013	-

[a] XRF. [b] N_2 sorption. [c] Volumetric CO_2 chemisorption. [d] Determined by normalization of the CO_2 uptake per m^2 respective to unpromoted In_2O_3.

Supplementary Table 5. Abundance of In_2O_3 terminations determined by fringe analysis of HRTEM images of fresh 1Ni-In_2O_3.

Termination	Count [-]	Frequency [%]
(222)	14	61
(044)	4	18
(004)	3	13
(226)	1	4
(033)	1	4

Supplementary Table 6. Surface coverage by nickel, nickel content on In_2O_3 in weight percent, and number of nickel atoms adsorbed on a $In_2O_3(111)$ cell. The surface areas of *x*Ni-In_2O_3 catalysts assumed to be 72 m^2 g^{-1} for all calculations. The area of $In_2O_3(111)$ $p(1\times1)$ is 183.55 $Å^2$, while the area occupied by a nickel atom in Ni(111) is 5.36 $Å^2$.

System	Coverage monolayers	Ni content [wt.%]	Number of nickel atoms
15Ni-In_2O_3	1.18	15.0	40.3
10Ni-In_2O_3	0.79	10.0	27.0
5Ni-In_2O_3	0.39	5.0	13.4
1Ni-In_2O_3	0.08	1.0	2.7
Nickel monolayer	1.00	12.7	34.2
Ni_{36}-In_2O_3	1.05	13.4	36.0
Ni_{25}-In_2O_3	0.73	9.3	25.0
Ni_5-In_2O_3	0.15	1.9	5.0
Ni_4-In_2O_3	0.12	1.5	4.0
Ni_3-In_2O_3	0.09	1.1	3.0
Ni_2-In_2O_3	0.06	0.7	2.0
Ni_1-In_2O_3	0.03	0.4	1.0

Supplementary Table 7. Metal and oxide bond energies (E_{bond}) of nickel and indium systems.

Bond	ΔE_{bond} [eV]
Ni-Ni	−0.86
In-In	−0.47
Ni-In	−0.93
Ni-O	−1.58
In-O	−1.87

Supplementary Table 8. Cohesive (ΔE_c) and formation (ΔE_f) energy of metals, metal oxides, and intermetallics. Formation energies are calculated with respect to isolated gas-phase metal atoms for bulk metals, to isolated gas-phase metal atoms and O_2 for metal oxides, and as the energy to form the alloy and H_2O from the metal oxide carrier, isolated gas-phase metal atoms, and O_2 for intermetallics. They are reported by metal atom for metal oxides and whereas by the number of deposited metal atoms for intermetallics. References to crystallographic structures are included when needed. Structural types are indicated between parentheses.

System	ΔE_c [eV]	ΔE_f [eV]	Ref.
Ni (face-centered cubic, fcc)	4.84	−4.84	-
Pd (fcc)	3.70	−3.70	-
Pt (fcc)	5.53	−5.53	-
Cu (fcc)	3.48	−3.48	-
Ce (fcc)	4.18	−4.18	-
Zn (hexagonal closed-packed, hcp)	1.10	−1.10	-
Ti (hcp)	3.63	−3.63	-
In (body-centered tetragonal)	2.35	−2.35	-
In_2O_3 (bixbyite)	22.82	−6.37	9
CeO_2 (fluorite CaF_2)	20.40	−13.68	10
TiO_2 (rutile RuO_2)	19.82	−13.09	11
ZnO (wurtzite, ZnS)	7.37	−4.01	12
PtO (PdO)	9.39	−6.03	13
PtO_2 ($CaCl_2$)	13.68	−6.96	14
Cu_2O (Cu_2O)	11.54	−4.09	15
CuO (CuO mS8)	8.02	−4.66	15
NiO (NaCl)	9.16	−5.80	16
Pd_2O (Cu_2O)	11.24	−3.94	17
PdO (PtS)	7.97	−4.61	18
PdO_2 (rutile TiO_2)	11.25	−4.53	19
InNi (CoSn)	7.41	−4.83	20
InNi (CsCl)	7.59	−5.01	20
$InNi_2$ (Co_2Ge)	12.15	−4.79	21
$InNi_3$ (Cu_3Au)	17.09	−4.84	20
$InNi_3$ (Ni_3Sn)	17.20	−4.87	20
In_3Ni_2 (Ni_2Al_3)	17.62	−4.94	20
In_9Ni_{13} (In_9Pt_{13})	87.05	−4.91	22
InPd (CsCl)	7.06	−4.48	23

Table continued on next page

System	ΔE_c [eV]	ΔE_f [eV]	Ref.
InPd (CsCl)	7.06	−4.48	23
$InPd_2$ (Co_2Si)	11.32	−4.37	24
$InPd_3$ ($LiPd_2Tl$)	15.29	−4.24	25
$InPd_3$ ($ZrAl_3$)	15.32	−4.25	26
In_3Pd_2 (Ni_2Al_3)	16.86	−4.56	27
In_3Pd_5 ($SnLa_2S_5$)	29.83	−4.42	24
In_7Pd_3 (Ru_3Sn_7)	31.52	−4.49	28
CePt (TiI)	12.02	−3.39	29
$CePt_2$ (Laves $MgCu_2$)	19.08	−5.23	30
$CePt_3$ (Cu_3Au)	25.47	−5.61	31
$CePt_5$ ($CaCu_5$)	36.90	−5.66	32
Ce_3Pt_4 (Pu_3Pd_4)	44.71	−4.71	33
Ce_7Pt_3 (Th_7Fe_3)	57.88	0.84	34
ZnCu (CsCl)	4.84	−3.28	35
Zn_8Cu_5 (Cu_5Zn_8)	27.94	−3.14	36
TiPt (AuCd)	10.20	−2.16	37
TiPt (CsCl)	10.00	−1.96	38
Ti_3Pt (Cr_3Si)	16.51	7.63	39
$TiPt_8$ ($TiPt_8$)	51.02	−5.37	40
$TiPt_3$ (Cu_3Au)	22.83	−4.93	41
$TiPt_3$ ($TiNi_3$)	22.82	−4.92	42
Ti_3Pt_5 (Ti_3Pt_5)	43.66	−3.90	43

Supplementary Table 9. Calculated formation energy and experimental standard enthalpy formation with respect to either gas-phase isolated metals ($\Delta E_{f\text{-gas}}$, $\Delta H_{f\text{-gas,exp}}^{0}$) or bulk-phase metal ($\Delta E_{f\text{-bulk}}$, $\Delta E_{f\text{-bulk,exp}}$) and O_2(g). Data for ΔH^{0}_{exp} are collected from ref. 44.

System	$\Delta E_{f\text{-gas}}$ [eV]	$\Delta H_{f\text{-gas, exp}}^{0}$ [eV]	$\Delta E_{f\text{-bulk}}$ [eV]	$\Delta H_{f\text{-bulk, exp}}^{0}$ [eV]
Ni (fcc)	−4.84	−4.45	0.00	-
Pd (fcc)	−3.70	−3.92	0.00	-
Pt (fcc)	−5.53	−5.86	0.00	-
Cu (fcc)	−3.48	−3.50	0.00	-
Ce (fcc)	−4.18	−4.38	0.00	-
Zn (hcp)	−1.10	−1.35	0.00	-
Ti (hcp)	−3.63	−4.90	0.00	-
In (bct)	−2.35	−2.52	0.00	-
In_2O_3 (bixbyite)	−12.73	-	−8.04	−9.60
CeO_2 (fluorite CaF_2)	−13.68	-	−9.49	−11.30
TiO_2 (rutile RuO_2)	−13.09	-	−9.47	−9.78
ZnO (wurtzite, ZnS)	−4.01	-	−2.91	−3.63
PtO (PdO)	−6.03	-	−0.50	−0.74
PtO_2 ($CaCl_2$)	−6.96	-	−1.43	−1.39
Cu_2O (Cu_2O)	−8.18	-	−1.23	−1.75
CuO (CuO mS8)	−4.66	-	−1.18	−1.63
NiO (NaCl)	−5.80	-	−0.97	−2.53
Pd_2O (Cu_2O)	−7.88	-	−0.48	-
PdO (PtS)	−4.61	-	−0.90	−0.89
PdO_2 (rutile TiO_2)	−4.53	-	−0.83	-

Supplementary References

1 Chen, M. *et al.* Dehydrogenation of propane over In_2O_3–Al_2O_3 mixed oxide in the presence of carbon dioxide. *J. Catal.* **272**, 101-108 (2010).

2 Hsu, W.-N. *et al.* Deformation and degradation of superelastic NiTi under multiaxial loading. *Acta Mater.* **167**, 149-158 (2019).

3 Abdala, P. M. *et al.* Scientific opportunities for heterogeneous catalysis research at the SuperXAS and SNBL beam lines. *CHIMIA* **66**, 699-705 (2012).

4 Müller, O., Nachtegaal, M., Just, J., Lützenkirchen-Hecht, D. & Frahm, R. Quick-EXAFS setup at the SuperXAS beamline for in situ X-ray absorption spectroscopy with 10 ms time resolution. *J. Synchrotron Radiat.* **23**, 260-266 (2016).

5 Ravel, B. & Newville, M. ATHENA, ARTEMIS, HEPHAESTUS: Data analysis for X-ray absorption spectroscopy using IFEFFIT. *J. Synchrotron Radiat.* **12**, 537-541 (2005).

6 Fierro, J. L. G. & Delabanda, J. F. G. Chemisorption of probe molecules on metal-oxides. *Catal. Rev. - Sci. Eng.* **28**, 265-333 (1986).

7 Frei, M. S. *et al.* Atomic-scale engineering of indium oxide promotion by palladium for methanol production via CO_2 hydrogenation. *Nat. Commun.* **10**, 3377 (2019).

8 Tauster, S. J. Strong metal-support interactions. *Acc. Chem. Res.* **20**, 389-394 (1987).

9 Marezio, M. Refinement of the crystal structure of In_2O_3 at two wavelengths. *Acta Crystallogr.* **20**, 723-728 (1966).

10 Sharan, R. & Dutta, A. Structural analysis of Zr^{4+} doped ceria, a possible material for ammonia detection in ppm level. *J. Alloys Compd.* **693**, 936-944 (2017).

11 Baur, W. H. & Khan, A. A. Rutile-type compounds. IV. SiO_2, GeO_2 and a comparison with other rutile-type structures. *Acta Crystallogr., Sect. B* **27**, 2133-2139 (1971).

12 Abrahams, S. C. & Bernstein, J. L. Remeasurement of the structure of hexagonal ZnO. *Acta Crystallogr., Sect. B* **25**, 1233-1236 (1969).

13 Moore, W. J. & Pauling, L. The crystal structures of the tetragonal monoxides of lead, tin, palladium, and platinum. *J. Am. Chem. Soc.* **63**, 1392-1394 (1941).

14 Müller, O. & Roy, R. Formation and stability of the platinum and rhodium oxides at high oxygen pressures and the structures of Pt_3O_4, β-PtO_2 and RhO_2. *J. Less-Common Met.* **16**, 129-146 (1968).

15 Niggli, P. XII. Die Kristallstruktur einiger Oxyde. *Z. Kristallogr.* **57**, 253-299, (1922).

16 Barrett, C. A. & Evans, E. B. Solid solubility and lattice parameter of NiO-MnO. *J. Am. Ceram. Soc.* **47**, 533-533 (1964).

17 Kumar, J. & Saxena, R. Formation of NaCl- and Cu_2O-type oxides of platinum and palladium on carbon and alumina support films. *J. Less-Common Met.* **147**, 59-71 (1989).

18 Glemser, O. & Peuschel, G. Beitrag zur Kenntnis des systems PdO/H_2O. *Z. Anorg. Allg. Chem.* **281**, 44-53 (1955).

19 Shaplygin, I. S., Aparnikov, G. L. & Lazarev, V. B. Preparation of palladium dioxide at high-pressure. *Russ. J. Inorg. Chem.* **23**, 884-887 (1978).

20 Hellner, E. Das System Nickel-Indium. *Z. Metallkd.* **41**, 401-406, (1950).

21 Singleton, M. F. & Nash, P. The In-Ni (indium-nickel) system. *Bull. Alloy Phase Diagrams* **9**, 592-597 (1988).

22 Norén, L., Larsson, A. K., Withers, R. L. & Rundlöf, H. A neutron and X-ray powder diffraction study of $B8_2$ related superstructure phases in the Ni–In system. *J. Alloys Compd.* **424**, 247-254 (2006).

23 Erwin, H. & Fritz, L. Kristallchemie des In und Ga in Legierungen mit einigen Übergangselementen (Ni, Pd, Pt, Cu, Ag und Au). *Z. Naturforsch.* **2**, 177-184 (1947).

24 Holger, K. & Clemens, R. Refinement of the crystal structures of palladium-rich In-Pd compounds by X-ray and neutron powder diffraction. *Z. Naturforsch., B* **62**, 929-934 (2007).
25 Kohlmann, H. & Ritter, C. Reaction pathways in the formation of intermetallic $InPd_3$ polymorphs. *Z. Anorg. Allg. Chem.* **635**, 1573-1579 (2009).
26 Kohlmann, H. Hydrogenation of palladium rich compounds of aluminium, gallium and indium. *J. Solid State Chem.* **183**, 367-372 (2010).
27 Harris, I. R., Norman, M. & Bryant, A. W. A study of some palladium-indium, platinum-indium and platinum-tin alloys. *J. Less-Common Met.* **16**, 427-440 (1968).
28 Häussermann, U., Elding-Pontén, M., Svensson, C. & Lidin, S. Compounds with the Ir_3Ge_7 structure type: Interpenetrating frameworks with flexible bonding properties. *Chem. Eur. J.* **4**, 1007-1015 (1998).
29 Krikorian, N. H. The reaction of selected lanthanide carbides with platinum and iridium. *J. Less-Common Met.* **23**, 271-279 (1971).
30 Zachariasen, W. Crystal chemical studies of the 5f-series of elements. XII. New compounds representing known structure types. *Acta Crystallogr.* **2**, 388-390 (1949).
31 Moriarty, J. L., Jnr, Humphreys, J. E., Gordon, R. O. & Baenziger, N. C. X-ray examination of some rare-earth-containing binary alloy systems. *Acta Crystallogr.* **21**, 840-841 (1966).
32 Adroja, D. T., Malik, S. K., Padalia, B. D. & Vijayaraghavan, R. Structural and magnetic studies on $CeTPt_4$ (T = Cu, Ga, Rh, Pd, and In) compounds. *Solid State Commun.* **71**, 649-651 (1989).
33 Palenzona, A. The crystal structure and lattice constants of R_3Pt_4 compounds. *J. Less-Common Met.* **53**, 133-136 (1977).
34 Olcese, G. L. Crystal structure and magnetic properties of some 7:3 binary phases between lanthanides and metals of the 8th group. *J. Less-Common Met.* **33**, 71-81 (1973).
35 Rao, S. S. & Anantharaman, T. Constitution of brasses below 500°C. *Z. Metallkd.* **60**, 312-315 (1969).
36 Singh, P. & Hodgson, D. J. Aza analogs of nucleic acid constituents. IV. The crystal and molecular structure of 6-azauracil. *Acta Crystallogr., Sect. B* **30**, 1430-1435 (1974).
37 Donkersloot, H. C. & Van Vucht, J. H. N. Martensitic transformations in gold-titanium, palladium-titanium and platinum-titanium alloys near the equiatomic composition. *J. Less-Common Met.* **20**, 83-91 (1970).
38 Dwight, A. E., Conner, R. A., Jnr & Downey, J. W. Equiatomic compounds of the transition and lanthanide elements with Rh, Ir, Ni and Pt. *Acta Crystallogr.* **18**, 835-839 (1965).
39 Duwetz, P. & Jordan, C. B. The crystal stucture of Ti_3Au and Ti_3Pt. *Acta Crystallogr.* **5**, 213-214 (1952).
40 Pietrokowsky, P. Novel ordered phase, Pt_8Ti. *Nature* **206**, 291-291 (1965).
41 Dwight, A. E. & Beck, P. E. Close-packed ordered structures in binary AB_3 alloys of transition elements. *Trans. Metall. Soc. AIME* **215**, 237-240 (1959).
42 Schubert, K., Raman, A. & Rossteutscher, W. Einige Strukturdaten metallischer Phasen. *Naturwissenschaften* **51**, 506-507 (1964).
43 Krautwasser, P., Bahn, S. & Schubert, K. Strukturuntersuchungen in den Systemen Ti-Pd und Ti-Pt. *Z. Metallkd.* **59**, 724-729 (1958).
44 Haynes, W. M., Lide David R. & Bruno, T. J. *CRC Handbook of Chemistry and Physics: A Ready-Reference Book of Chemical and Physical Data*. 97 edn, (CRC Press, Florida, 2016).
