# Supplementary material for: Nanostructure of nickel-promoted indium oxide catalysts drives selectivity in CO2 hydrogenation
Source: Nat Commun. 2021 Mar 30;12:1960. doi: 10.1038/s41467-021-22224-x (PMC8010022; doi:10.1038/s41467-021-22224-x)
Supplement: Supplementary file 3 — Description of Additional Supplementary Files [file 41467_2021_22224_MOESM3_ESM.pdf]

Description of Additional Supplementary Files

File Name: Supplementary Movie 1
Description: Relaxation of a commensurate 5×5 Ni(111) layer on $In_2O_3(111)$, corresponding to the surface shown in Figure 4 of the main manuscript. Oxygen atoms stripped by Ni are shown in purple.
